# Time-Dependent Statistical and Correlation Properties of Neural Signals during Handwriting

**DOI:** 10.1371/journal.pone.0043945

**Published:** 2012-09-11

**Authors:** Valery I. Rupasov, Mikhail A. Lebedev, Joseph S. Erlichman, Stephen L. Lee, James C. Leiter, Michael Linderman

**Affiliations:** 1 Department of Basic Research, Norconnect Inc., Ogdensburg, New York, United States of America; 2 Department of Neurobiology, Duke University, Durham, North Carolina, United States of America; 3 Department of Biology, St. Lawrence University, Canton, New York, United States of America; 4 Department of Neurology, Dartmouth Medical School, Lebanon, New Hampshire, United States of America; 5 Department of Physiology and Neurobiology, Dartmouth Medical School, Lebanon, New Hampshire, United States of America; 6 Department of Neuroethics, Norconnect Inc., Ogdensburg, New York, United States of America; University Medical Center Groningen UMCG, The Netherlands

## Abstract

To elucidate the cortical control of handwriting, we examined time-dependent statistical and correlational properties of simultaneously recorded 64-channel electroencephalograms (EEGs) and electromyograms (EMGs) of intrinsic hand muscles. We introduced a statistical method, which offered advantages compared to conventional coherence methods. In contrast to coherence methods, which operate in the frequency domain, our method enabled us to study the functional association between different neural regions in the time domain. In our experiments, subjects performed about 400 stereotypical trials during which they wrote a single character. These trials provided time-dependent EMG and EEG data capturing different handwriting epochs. The set of trials was treated as a statistical ensemble, and time-dependent correlation functions between neural signals were computed by averaging over that ensemble. We found that trial-to-trial variability of both the EMGs and EEGs was well described by a log-normal distribution with time-dependent parameters, which was clearly distinguished from the normal (Gaussian) distribution. We found strong and long-lasting EMG/EMG correlations, whereas EEG/EEG correlations, which were also quite strong, were short-lived with a characteristic correlation durations on the order of 100 ms or less. Our computations of correlation functions were restricted to the 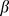 spectral range (13–30 Hz) of EEG signals where we found the strongest effects related to handwriting. Although, all subjects involved in our experiments were right-hand writers, we observed a clear symmetry between left and right motor areas: inter-channel correlations were strong if both channels were located over the left or right hemispheres, and 2–3 times weaker if the EEG channels were located over different hemispheres. Although we observed synchronized changes in the mean energies of EEG and EMG signals, we found that EEG/EMG correlations were much weaker than EEG/EEG and EMG/EMG correlations. The absence of strong correlations between EMG and EEG signals indicates that (i) a large fraction of the EEG signal includes electrical activity unrelated to low-level motor variability; (ii) neural processing of cortically-derived signals by spinal circuitry may reduce the correlation between EEG and EMG signals.

## Introduction

Since the first publication by D. Walter [1], the coherence method, developed for the analysis of stationary random data in linear systems (see, e.g., [2]), has been employed in hundreds of papers dealing with the analysis of neural signals such as EEGs and EMGs. In these publications, the level of coherence was used as a measure of coupling between the processes generating neural signals and of the functional association between neuronal structures [3]–[6].

This analysis of relationships between neural signals is based on computations of the coherence and phase of the two signals. For Fourier harmonics, 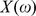 and 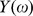, of two time-dependent signals 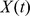 and 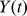, the coherence is defined as the square of the modulus, 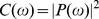, and the phase is defined as 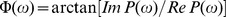, of the complex coherence function
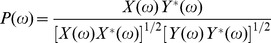
(1)Here, 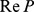 and 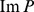 stand for the real and imaginary parts of the function 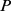.

Signals 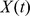 and 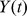 can also be sliced into 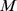 disjoint segments, 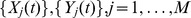, and the function 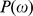 may be estimated as shown by Eq. (1) with auto- and cross-spectral density functions computed for each segment and averaged over 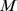 segments,
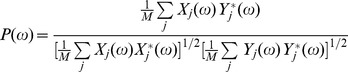
(2)


Coherence is considered to be statistically significant if its magnitude exceeds some value 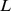. At any desired confidence level 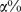 (in the most cases 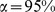), the confidence limit is estimated [7] under a Gaussian assumption as:
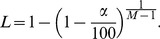
(3)A relatively small number of segments, 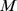, suffices to achieve any desired confidence limit due to the exponential dependence of the confidence limit on the number of segments.

Neural signals are obviously nonstationary. To address the nonstationarity of biological signals, the coherence method has been generalized [8] for wavelet harmonics [9]. Wavelet-based methods allow a combined frequency- and time-domain representation of nonstationary signals.

In this paper, we propose and discuss an alternative approach to search for dynamical relationships between neural signals [10]. In this method, which is broadly employed in statistics and, in particular, in statistical physics, a relationship between two random time-dependent signals 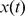 and 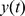 is determined by the correlation function
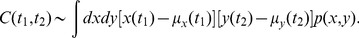
(4)Here 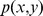 is the joint probability density function of two random variables, 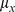 and 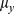 are the corresponding mean values 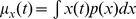 and 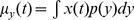, where 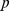 is the probability density function. It should be emphasized that for nonstationary systems, the time dependence of correlation functions is determined not only by the time dependencies of the signals themselves, but also by the time dependencies of the probability density functions.

For two independent random variables, the joint probability density function can be factorized, i.e., 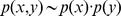, and the correlation function 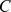 vanishes.

The probability density functions of neural signals are not known a priori. Therefore, one needs to have a sufficiently large set (statistical ensemble) of neural signals 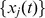 and 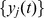, (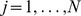) recorded during 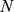 epochs - in our case, trials during which a subject repeatedly performs an identical task - in order to apply the statistical method. In this approach, the integration of probability density functions in Eq. (4) is replaced by the ensemble average over many trials,
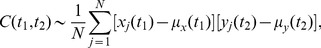
(5)where 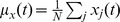 and 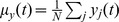 are the mean values.

This expression becomes exact only in the limit when the number of trials goes to infinity, 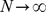. In our experiments the number of trials was about 400, and we used Fisher's theorem [11] to compute the confidence interval for the correlation functions.

Here we applied this statistical method to an analysis of EEG and EMG signals recorded during handwriting. Handwriting [12] provides an excellent neuromuscular task for quantitative studies of statistical and correlational properties of biological signals in the time domain. Handwriting consists of relatively simple, stereotyped hand movements that involve two basic motor components: firmly holding a pen by the fingers and moving the hand and the fingers to produce written text. We recorded the mechanical events of handwriting using a digitizing tablet that electronically recorded each epoch when the pen touched the paper (pen-on-paper period), and we simultaneously recorded the cortical EEG and EMG of intrinsic muscles of the hand.

Our dataset allowed us (i) to align the mechanical events, EEG signals and EMG signals associated with handwriting and (ii) to divide all of these signals into trial segments that correspond to a sequential set of epochs as each subject performed an identical handwriting task. Our trials started 1000 ms before the first moment when the pen touched the paper and ended 1000 ms after this time. This precise temporal sequence enabled us to compare statistical and correlation properties of neural signals recorded before, during and after the actual pen-on-paper activity.

The recorded data consisted of 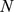 trials, each trial captured the activity associated with a single handwritten letter. Each trial contained EEGs recorded in 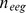 channels, and EMGs recorded in 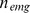 channels, i.e. in total, we obtained 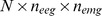 data files each of 2000 ms duration.

In contrast to the coherence methods used to study the relationship between neural signals in the frequency domain, our statistical method enabled us to study the statistical and correlational properties of neural signals directly in the time domain. That allows us to elucidate both dynamical patterns of activity in different cortical areas and the functional relationships between different cortical areas.

As in the case of EMG signals recorded from muscle groups involved in handwriting [13], we found that trial-to-trial variability of the “energy” of the EEGs recorded from the motor cortex area had a log-normal distribution, which was clearly distinguishable from the normal (Gaussian) distribution. The log-normal distribution fitted the experimental data in all time intervals during a trial, but its parameters - the mean value and dispersion - depended on time. These two variables suffice to completely describe both qualitatively and quantitatively the trial-to-trial variability of neural signals during handwriting.

We also studied time-dependent EMG/EMG and EEG/EEG correlations. We found strong and long-time EMG/EMG correlations. Correlations between EEG signals over the motor cortex area were also quite strong, but the correlations existed over relatively short durations with characteristic correlation times on the order of 100 ms or less. All subjects involved in our experiments were right-handed and wrote with their dominant hand. However, we observed equally strong inter-channel correlations when both channels were located on either the left or right hemispheres, while inter-hemispheric correlations were 2–3 times weaker.

Although we observed task-related changes in the mean energies of both EEG and EMG signals, we found that EEG/EMG correlations were much weaker than EEG/EEG and EMG/EMG correlations. In other words, trial-to-trial variations of the magnitudes of the EEG and EMG changes were related only weakly.

## Methods

This study was approved by the Institutional Review Board of Human Participants Research of Dartmouth College, Hanover, NH. No personal information was recorded during the sessions, and all data were analyzed anonymously. Written informed consent was obtained from the subjects prior to the recording sessions.

**Figure 1:**
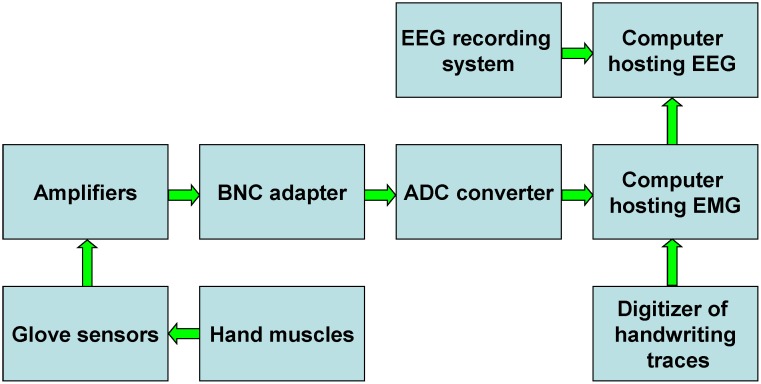
Block diagram of the experimental setup.

**Figure 2:**
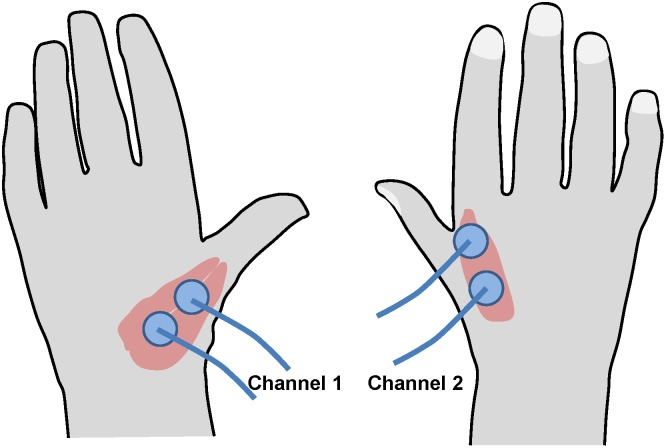
Placement of EMG sensors on the hand.

A schematic of the experimental setup for simultaneous recording EMG and EEG signals during handwriting is shown in **Figure 1**. We used LogiPen to record handwriting characters. EMG signals were sampled using bipolar surface EMG electrodes (Kendall Arbo). We used EMG analog amplifiers with gain 1000, low pass filter of 450 Hz, and high pass filter of 12 Hz. The EMG signals were digitized at a sample rate of 1000 Hz. The surface EMGs of the intrinsic hand muscles were recorded by two electrode pairs. One pair recorded EMG activity from flexor pollicis brevis and abductor pollicis brevis. The second pair recorded from the first dorsal interosseus muscle (**Figure 2**). EEG activity was sampled with a standard 64-channel device. The data from the 64-channel EEG recording system were high-pass filtered with 8 Hz cutoff, digitized at 1000 Hz and saved on a computer. Handwriting and EMG inputs were synchronized by a LabView data acquisition program based on the pen-down event associated with each handwriting trial. We used LogiManage as the digitizer of handwriting traces. It recorded X and Y coordinates, as well as the pen-on-paper signal. EMG signals were recorded on a dedicated computer that was also connected to the EEG recording system via a parallel port. The pen-on-paper signals were used to align EEG and EMG recordings.

To carry out quantitative studies of time-dependent statistical and correlation properties of neural signals, we instructed seven subjects to write the digit “3”. This task produced consistent motor patterns with some trial-to-trial variability. The single character was written about 400 times in 10 blocks of 40 trials. The blocks were separated by 5 minute rest intervals.

Handwriting trials were defined as the epochs starting 1000 ms before the time moment when the pen touches the tablet at the onset of writing a particular character and ending 1000 ms later. In all cases, the character was completely written before the end of the trial. After filtering the signals, their amplitudes, 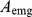 and 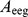, containing frequencies from 8 to 450 Hz, were squared to get the signal “intensities” 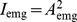 and 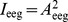.

To study time-dependent statistical and correlation properties of the signals, 2000-ms trials were subdivided into 20 time intervals, each with a duration of 100 ms, and the signal “energy” was calculated for each of 20 intervals as the sum,
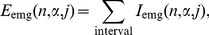
(6a)

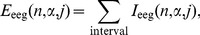
(6b)where 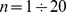, 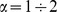 for EMG signals and 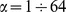 for EEG signals, and 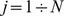 (where 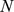 is the total number of trials), enumerate the time intervals, recording channels, and trials, respectively.

The minimal length of the time intervals was dictated by the accuracy with which trials could be aligned with respect to each other. In our experiments, the accuracy of alignment did not exceeded a few tens of millisecond, which was determined by the accuracy of the digitizing tablet used to detect the first moment of time when the pen touches the paper. Therefore, we chose a 100-ms time interval. This interval substantially exceeds the temporal resolution of the digitizing tablet and ensures an accurate trial alignment.

To obtain dimensionless variables for each interval and trial, the energies 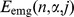 and 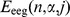 were normalized by dividing by their mean values 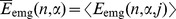 and 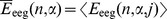. Where the symbol 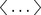 stands for averaging over trials,
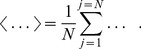
(7)Thus, the EMG and EEG signals for each recording channel 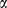 and each trial 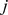 were characterized by dimensionless energies
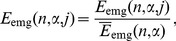
(8a)

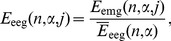
(8b)and their time dependence was described by the discrete variable 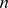 (time interval number).

## Results

### 1. Statistics of EMG signals

We previously studied the statistical properties of EMG signals during handwriting [13]. The results of this previous study are summarized in this Section for the convenience of readers and for further comparison with the properties of the EEG signals. Correlations properties of EMG signals, which have not studied earlier, are discussed in the next Section.

#### 1.1. Mean values and variation coefficients

**Figure 3:**
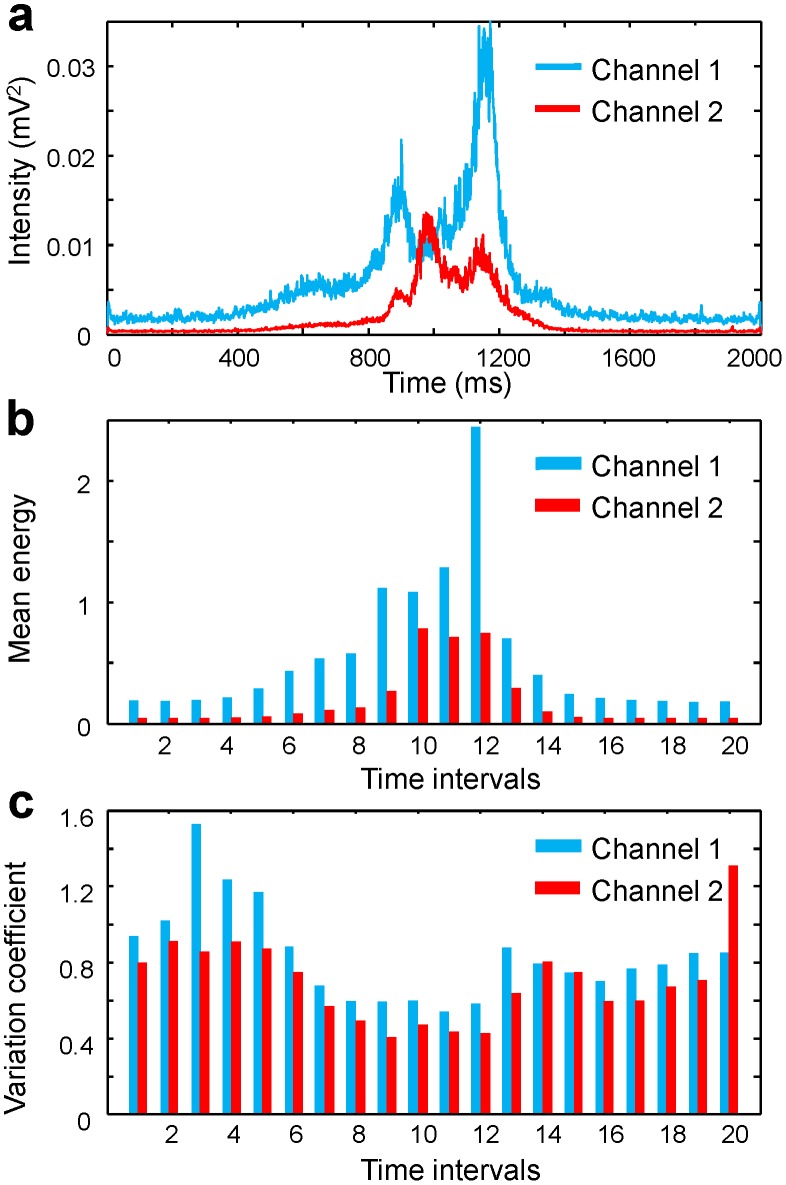
Statistical properties of EMG signals. (a) Average EMG intensities (EMG templates) calculated from 394 trials during which a subject wrote digit “3”. The point 1000 ms on the time axis corresponds to the initial moment of time when the pen touches the paper. (b) The mean energies of the EMG signals for 100 ms time intervals. The time interval 10 corresponds to the first 100 ms after the pen touched the paper. (c) The variation coefficients of EMG energy in each time interval.

Trial-averaged intensities of raw EMG signals, or EMG templates, 
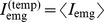
 described the characteristic pattern of EMG activity for the handwritten character for each of two muscle groups (**Figure 3a**). The time dependency of the mean energies 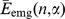 is shown in **Figure 3b**. The first 9 time intervals represent the epoch preceding the first pen touch. This subject wrote the digit “3” with a mean duration of the pen-on-paper period (i.e., time during which the pen touched the paper) of about 600–700 ms, which corresponds to time intervals 10 through 15 or 16, while the intervals 17 through 20 correspond to the time period following the pen liftoff from the paper.

The variation coefficients, 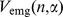, which characterize the dispersion of the data distribution, were computed as the ratio of the standard deviation
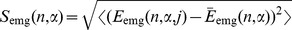
(9a)to the mean energy,
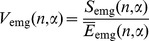
(9b)The variation coefficients were large for all time intervals and for both EMG channels (**Figure 3c**) and ranged from about 0.3 to 1. Thus, there was a substantial dispersion of EMG signals from trial to trial, and the EMG activity during handwriting could not be adequately described in terms of mean values only. More detailed analysis of the statistical properties of the EMG signals was required.

It should be noted that while the mean energies increased during the pen-on-paper period, the dispersion decreased. EMG patterns were more consistent when the subject wrote characters than when he/she lifted the pen off the paper. This pattern of the mean-energy and dispersion was observed for all subject, characters, and muscles.

#### 1.2. Distribution functions

**Figure 4:**
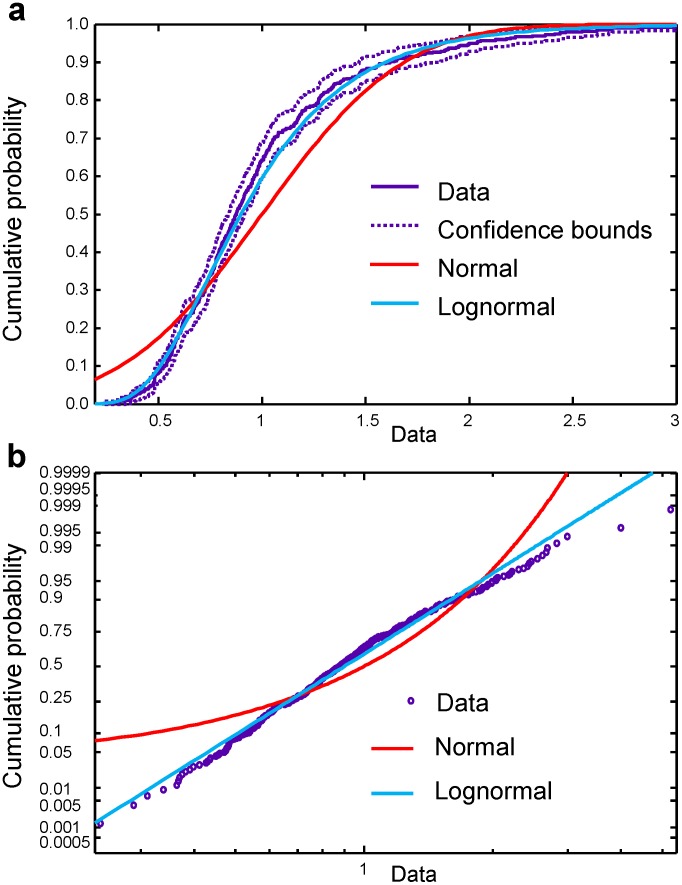
Probability plots for EMG signals. (a) Plot of empirical cumulative distribution function (time interval 11; EMG channel 1) together with theoretical cumulative distribution function plots for normal and log-normal distributions. The confidence bounds are shown for the confidence level of 95%. (b) Probability plot for experimental data for time interval 11, channel 1 together with probability plots for theoretical normal and log-normal distributions. Axis scales are chosen to have a straight line for the theoretical probability plot with the log-normal distribution.

We recorded a sufficiently large number of trials to approximate the theoretical probability distribution from our data. In **Figure 4**, the probability plots for experimental data (with no trial selection) are shown together with probability plots for the theoretical normal and log-normal distributions. It is clear that the experimental data are fitted well with the log-normal probability density function
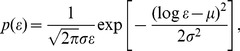
(10)where 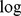 stands for natural logarithm.

Moreover, it is clear from **Figure 4** that the fitted curves for log-normal and normal distributions are easily distinguished, and that the log-normal distribution fits the data much better than the normal one.

### 2. Correlation functions of EMG signals

We observed that activation patterns of different muscles were coordinated during handwriting and the EMG bursts occurred at consistent interburst intervals. Mathematically, this relationship can be described by statistical correlation functions as described in the Introduction.

Since the logarithms of the random dimensionless energies (8a),
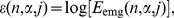
(11)are normally distributed across trials for each time interval, we may assume that the joint probability density function 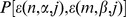 for pairs of the logarithms of dimensionless energies is the joint (second order) normal probability density function [2].

This allows one to compute both the correlation functions
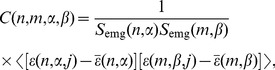
(12)where 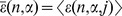, and, using the Fisher transform [11], to estimate the confidence intervals for the correlations. The Pearson correlations functions, defined in Eq. (12), are normalized to the standard deviations; therefore, their moduli do not exceed 1. We have described those correlation coefficients that approached 1 and were statistically significant as “strong” and those that approached 0 as “weak” correlations.

**Figure 5:**
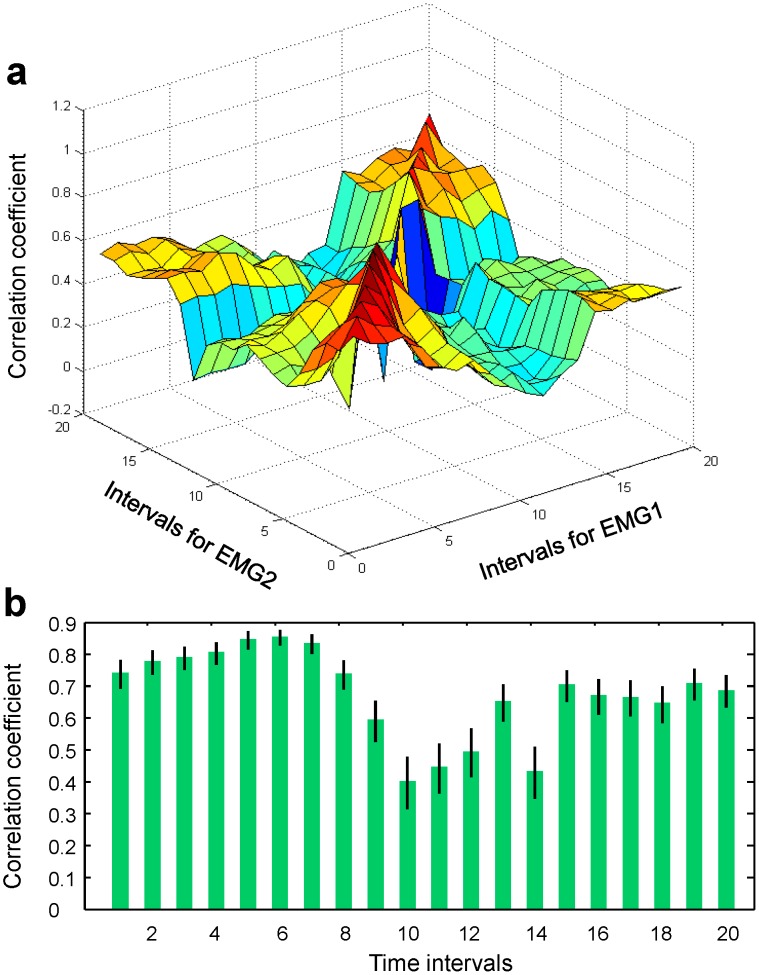
EMG/EMG correlation functions. (a) The cross-correlation function for EMG channels 1 and 2 (EMG1 and EMG2) as a function of two times: intervals for EMG1 and intervals for EMG2. (b) The cross-correlation function for EMG1 and EMG2 for coincident time intervals. Error bars indicate 95% confidence bounds.

**Figure 5** shows the cross-correlation function 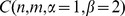 for two groups of muscles (EMG channels 1 and 2) involved in handwriting as a function of two times (**Figure 5a**) and the diagonal elements of this function at coincident time intervals 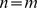 (**Figure 5b**). It is easy to see strong and long-time correlations between activities of muscle groups (**Figure 5a**) and particularly strong correlations at coincident time intervals. Correlations were higher outside the pen-on-paper period, when the subjects held the pen in the air, and decreased shortly before and during the pen-on-paper period.

**Figure 6:**
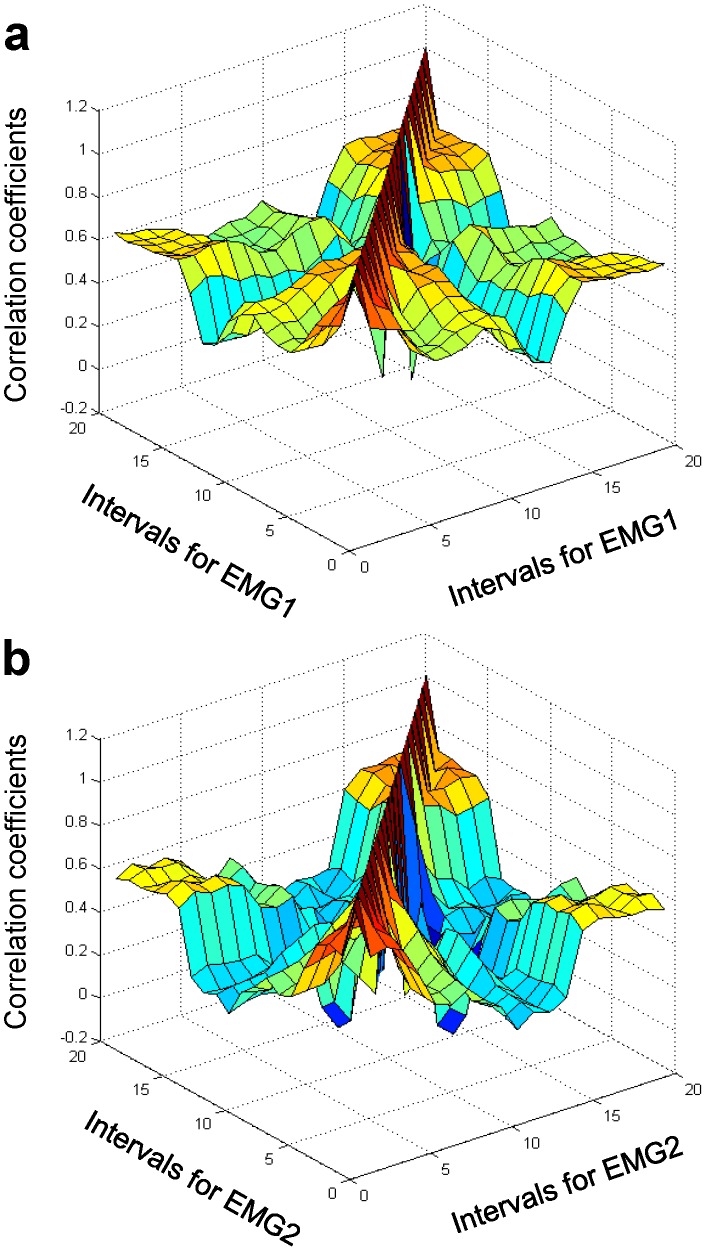
EMG/EMG correlation functions. (a) EMG1/EMG1 correlation function (i.e., EMG1 auto-correlation function). (b) EMG2 auto-correlation function.

Finally, autocorrelation functions, 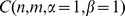 and 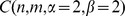 for both EMG signals are shown in **Figure 6**. Here again, strong correlations are clear over long time for each muscle group.

### 3. Statistical properties of EEG signals

As can be seen from the EMG templates, which indicate distinct bursts of activity (**Figure 3a**), and from the time-dependency of the EMG energy (**Figure 3b**), muscle activity was clearly patterned during the handwriting of the character. EEG activity even in the motor cortex area should not be expected to be limited to the handwriting itself and can contain other components that are not necessarily related to handwriting. Therefore, the total amplitude of EEG signal reads as
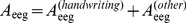
(13)and the non-handwriting contribution to the EEG obviously obscures the handwriting related component.

If a signal with amplitude 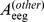 is unrelated to handwriting trials, averaging over many trials should reduce its contribution to the mean values and correlation functions. Nevertheless, since we square the total amplitudes of EEG signals, the contribution of the non-handwriting activity does not vanish completely.

**Figure 7:**
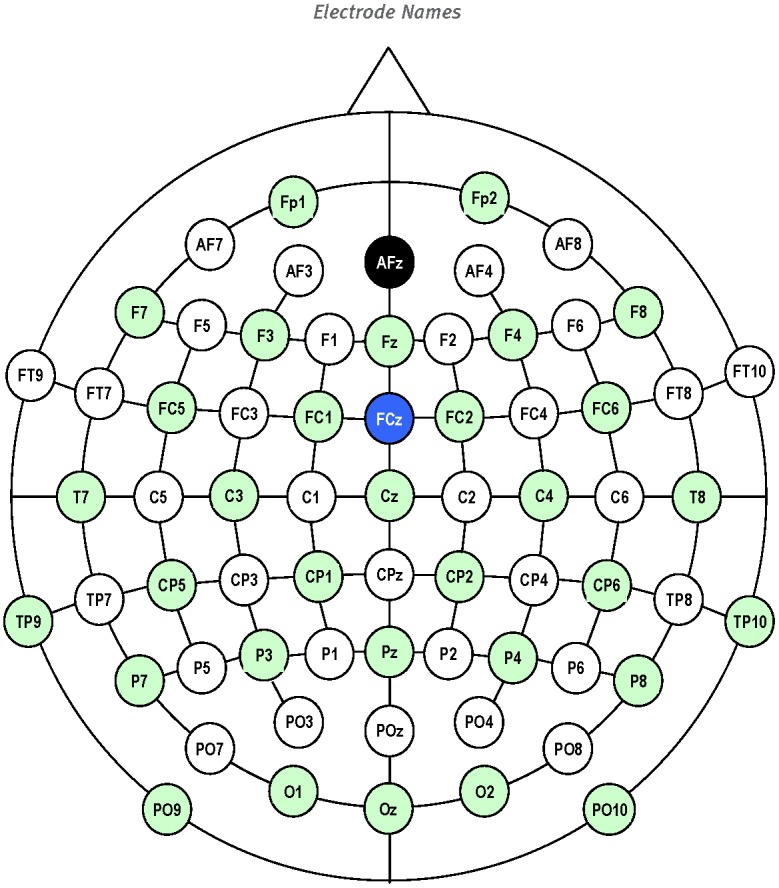
Surface map of EEG electrode locations.

To indicate each EEG channel, we used conventional electrode names shown in **Figure 7**.

#### 3.1. Mean values and variation coefficients

**Figure 8:**
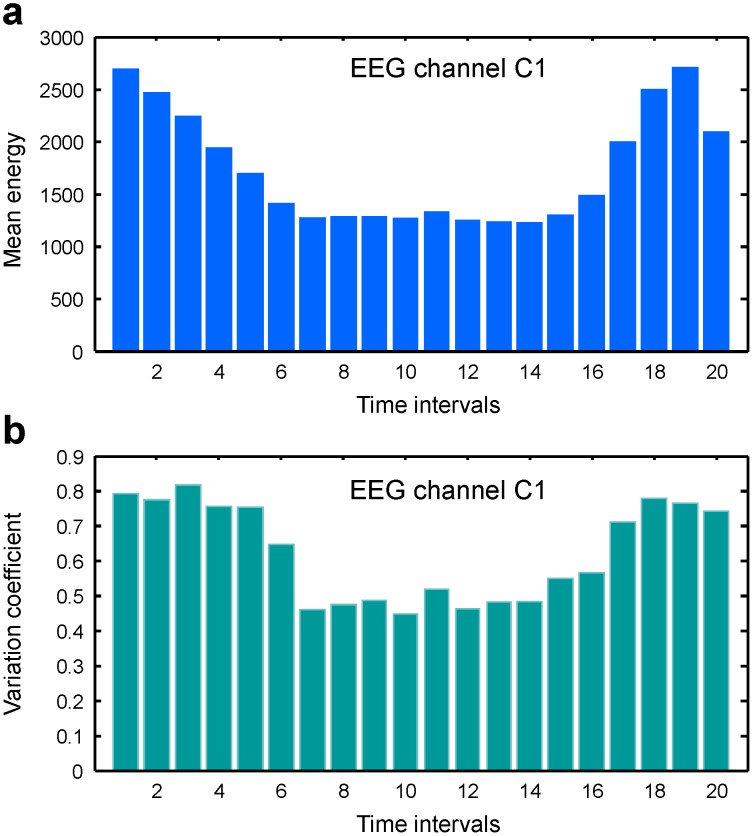
Statistical properties of raw EEG signals. (a) Time dependence of mean EEG energy on channel C1. (b) Variation coefficients for EEG energy on channel C1.

In contrast to EMG signals, we did not find prominent bursts in the EEG templates 
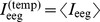
 for any of 64-channels that could be attributed to handwriting activity. **Figure 8a** represents the time behavior of the mean energy of EEG signal, 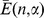, of channel C1, which was located over the left hemisphere.

The mean energy and variation of the EEG signal decreased about 300–400 ms before the pen-on-paper period and remained low during the actual period of writing. A similar time dependency of the mean energy was observed for channels FC5, FC3, FC1, C5, C3, CP5, CP3, and CP1 over the left hemisphere. It should be noted that during the 300-ms time period preceding the pen-on-paper period, EMG activity was increased (see **Figure 3a** and **Figure 3b**).

Thus, we found good correspondence between changes in EMG activity and EEG activity (**Figure 8a**) over the motor cortex. Even though all subjects involved in our studies were right handed, similar EEG changes were observed for the midline channels Cz and CPz, and the channels FC2, FC4, FC6, C2, C4, C6, CP2, CP4, and CP6 corresponding to the right hemisphere, locations that were mirror images of the channels with synchronized EMG/EEG activity over the left hemisphere.

As in the case of EMG signals, we defined the variation coefficient as a ratio of the standard deviation,
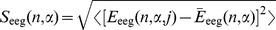
(14a)to the mean energy,
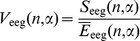
(14b)As in the case of EMG signals, the variation coefficients decreased shortly before and during the pen-on-paper period. Since the variation coefficients were quite large (**Figure 8b**), EEGs during handwriting could not be adequately described in terms of mean values only, and more detailed studies of their statistical properties were required.

**Figure 9:**
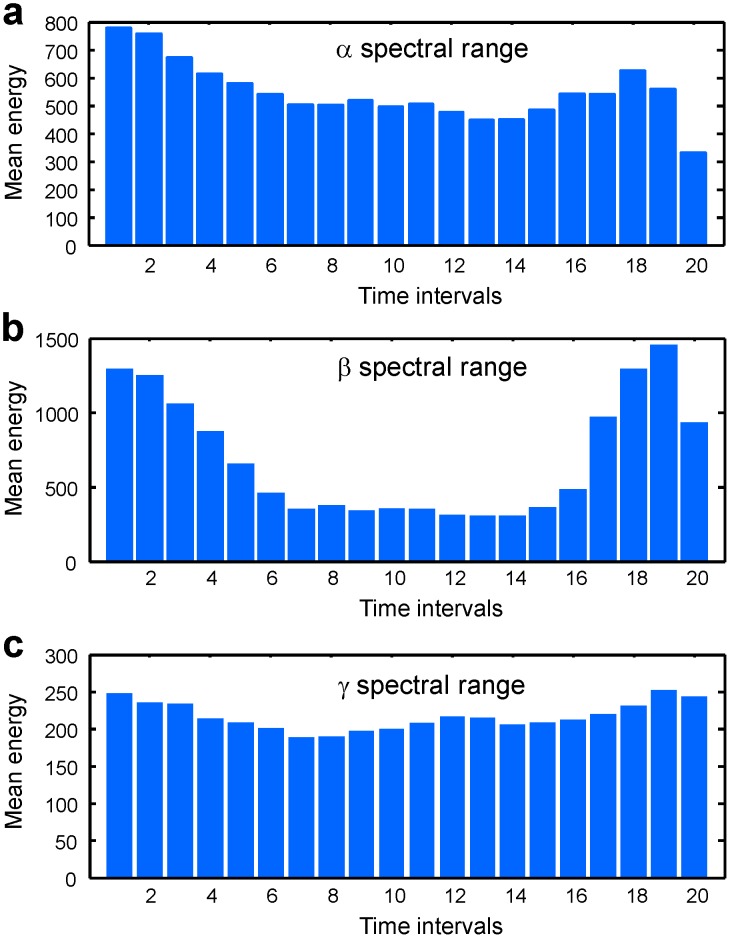
Statistical properties of EEG signals in different spectral ranges. Time-dependence of mean EEG energy on channel C1 in alpha (a), beta (b), and gamma (c) spectral ranges.

The relative magnitude of the EEG energy decrease during the pen-on-paper period was variable from subject to subject. It was maximal in the 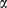 (8–13 Hz) and, especially, 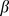 (13–30 Hz) spectral ranges and less apparent in the 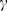 (30–100 Hz) spectral range (**Figure 9**).

It should be emphasized that the variation coefficients in the 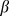 spectral range, where we observed the largest relative decrease in the mean EEG energies, increased quite significantly (up to two times) in comparison to other spectral ranges.

#### 3.2. Distribution functions

**Figure 10:**
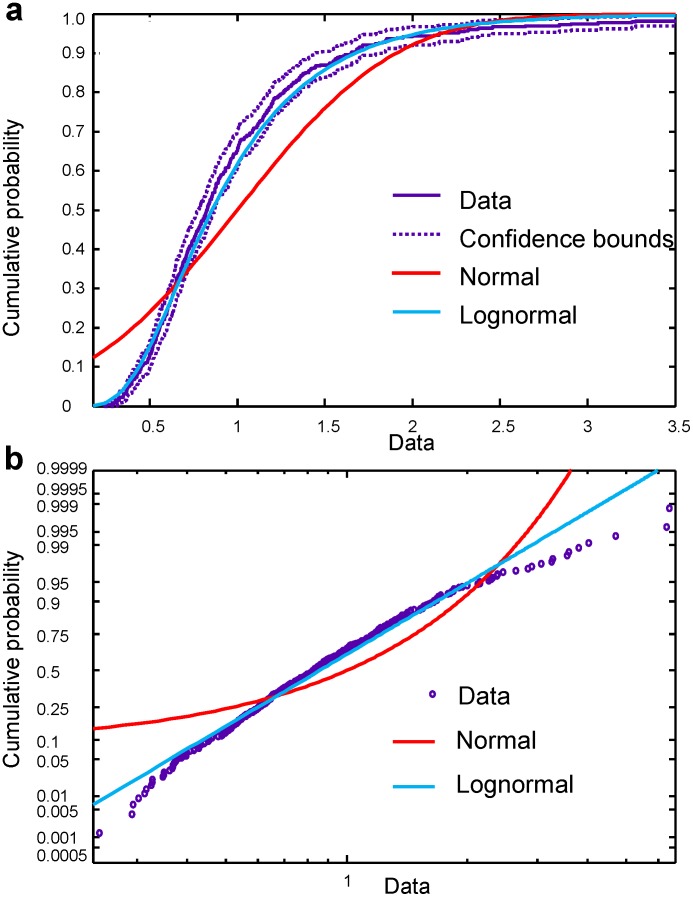
Probability plots for EEG signals. (a) Plot of empirical cumulative distribution function for the EEG on channel C3, time interval 10, together with the theoretical cumulative distribution function plots for normal and log-normal distributions. The confidence bounds correspond to a 95% confidence level. (b) Probability plot for experimental data together with probability plots for theoretical normal and log-normal distributions. The axis scales are chosen to make the theoretical probability plot of the log-normal distribution a straight line.

**Figure 10** shows that, as in the case of EMG signals, the theoretical log-normal function fits our EEG data better than a normal distribution. The theoretical log-normal cumulative distribution function lies inside the confidence bounds computed with the confidence level of 95%. Similar results were found in all time intervals for the EEG channels corresponding to the motor cortex in both left and right hemispheres. Some small deviations observed in some of the time intervals could be attributed to a contribution of the component of EEG activity not associated with handwriting activity, 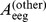.

Thus, the log-normal distribution effectively approximated the trial-to-trial variability of both EMG and EEG energy.

Since the relative magnitude of the EEG energy decrease during the pen-on-paper period was maximal in the 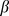 spectral range, we restricted our computations of correlation functions for EEG signals to this spectral range.

### 4. Correlation functions of EEG signals

Since logarithms of dimensionless energies of EEG signals, including energies of EEG signals in the 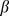 spectral range,
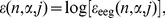
(15)were normally distributed, it was appropriate to compute the Pearson correlation functions as in the case of EMG signals, as
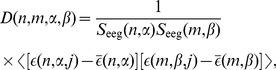
(16)where 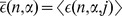.

First, we examined the correlation function at coincident time intervals, i.e. the diagonal elements 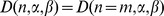. To study the spatial distribution of the EEG/EEG correlations for the motor cortex area, it was convenient to average the function 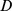 over the time intervals,
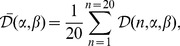
(17)and to study the averaged correlation coefficients 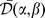 between EEG channels. This was possible because the EEG correlation functions did not vary greatly over time.

While decreases in the energy of EEG signals before and during the pen-on-paper period were observed (**Figure 9a**) over the motor cortex for channels from both the left (FC5, FC3, FC1, C5, C3, C1, CP5, CP3, and CP1) and right (FC2, FC4, FC6, C2, C4, C6, CP2, CP4, CP6) hemispheres, we found quite a different behavior for the correlation coefficients. Correlations were strong between channels located within the left and right parts of cortex and much weaker for channels located in opposite hemispheres. Moreover, correlations between the central channels (Cz and CPz) and channels on the left and right sides were also weak, despite the similar decreases in the EEG energy in these channels before and during the pen-on-paper period.

**Figure 11:**
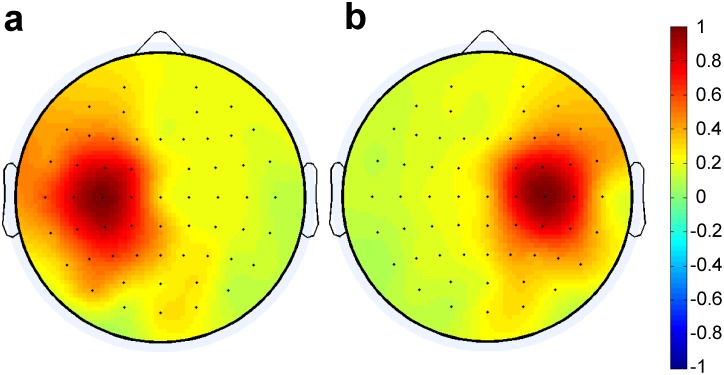
Color-coded maps for the EEG/EEG correlations. (a) Correlation coefficients between channel C3 and all other EEG channels. (b) Correlation coefficients between channel C4 and all other EEG channels.

The left-right symmetry of the cortex activity during handwriting is seen most clearly from the “color-coded maps” (**Figure 11**), where the magnitudes of correlation coefficients are shown by color.

**Figure 12:**
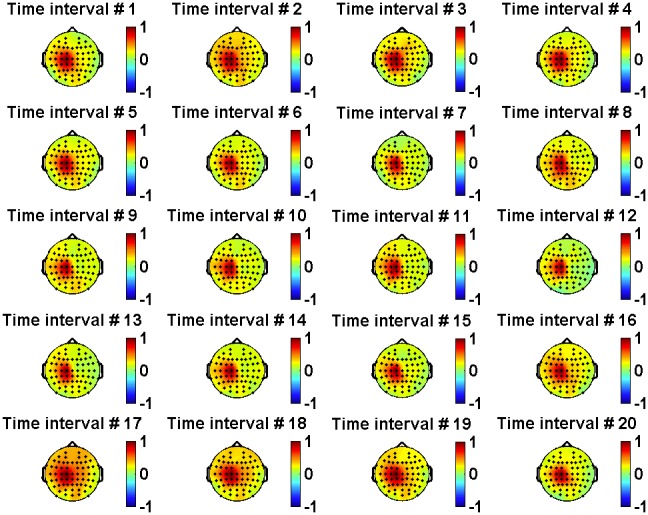
Changes in EEG/EEG correlations during the trial. Color-coded plots show correlation coefficients between channel C1 and all other EEG channels for consecutive time intervals.

**Figure 13:**
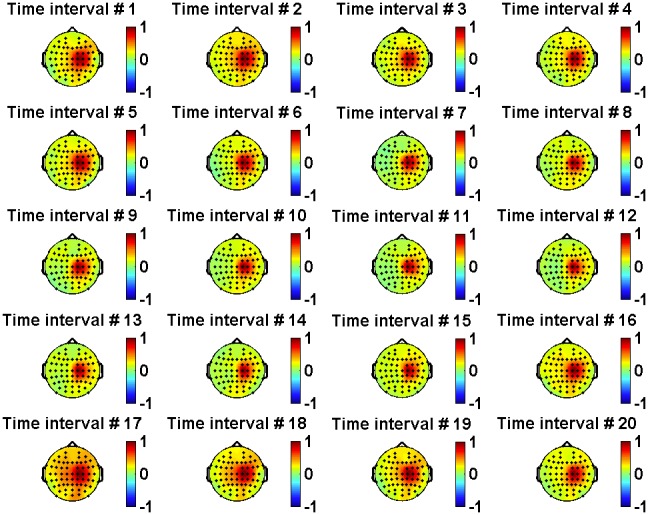
Changes in EEG/EEG correlations during the trial. Color-coded plots show correlation coefficients between channel C2 and all other EEG channels for consecutive time intervals.

The statistical method enabled us to easily derive the correlation coefficients for each time interval to get a dynamical picture of functional connectivity between different neural regions of the cortex during handwriting (see **Figure 12** and **Figure 13**).

**Figure 14:**
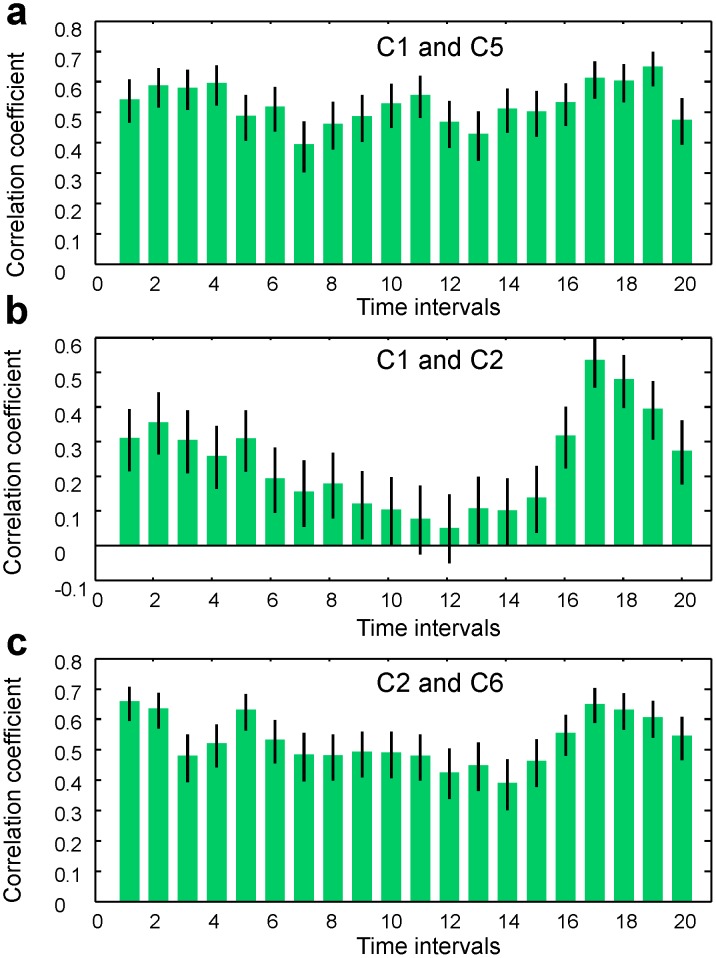
Time-dependent EEG/EEG correlation functions for three pairs of channels. Error bars indicate 95% confidence bounds. (a) Channels C5 and C1 - both located over the left hemisphere. (b) Channels C1 and C2 - C1 over the left and C2 over the right hemisphere. (c) Channels C2 and C6 - both located over the right hemisphere.

As seen in **Figure 11**, **Figure 12** and **Figure 13**, the correlation magnitudes were not determined only by the spacing between channels. However, the origin of surface EEG signals is not truly localized and discrete. There can be a problem of “volume conduction” in which any given EEG signal recorded by an electrode may contain components created by neural activity in areas located under other (neighboring) electrodes. To address this issue, we analyzed the time-dependent correlation functions at coincident time intervals for three pairs of channels: C5/C1 (both located on the left hemisphere), C1/C2 (located on different hemispheres), and C2/C6 (both located on the right hemisphere). This analysis is presented in **Figure 14**. Although the inter-electrode distances in all three pairs were approximately equal to each other, we observed that inter-hemispheric correlations were at least 2–5 times lower than intra-hemispheric ones for time intervals before and during handwriting. Thus, volume conduction appears to have a small impact on the correlations that we computed based on the ensembles of identical trials.

**Figure 15:**
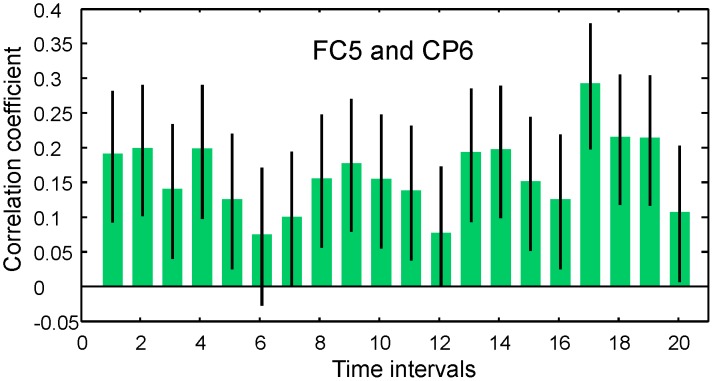
EEG/EEG correlation function between channels FC5 and CP6 with the maximal inter-channel spacing over the motor cortex area. Error bars indicate 95% confidence bounds.

Moreover, in **Figure 15** we present the correlation function between EEG signals recorded in channels FC5 and CP6. We observed time-dependent and statistically significant correlations on the order of 0.2–0.3 despite the maximally large (see **Figure 7**) spatial separation between electrodes located in the motor cortex area with the inter-electrode spacing, 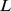, about of 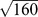 cm. The contribution of the dipole electric field generated by the cortex located under the electrode FC5 to the electrical potential recorded by the electrode CP6 was estimated in the simplest way as 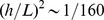, where 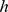 (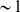 cm) is the distance between the electrode and cortex. Thus, the contribution of the electrical field generated at FC5 to activity at CP6 was very small, and the correlation between these EEG signals cannot be attributed to the volume conduction effect.

**Figure 16:**
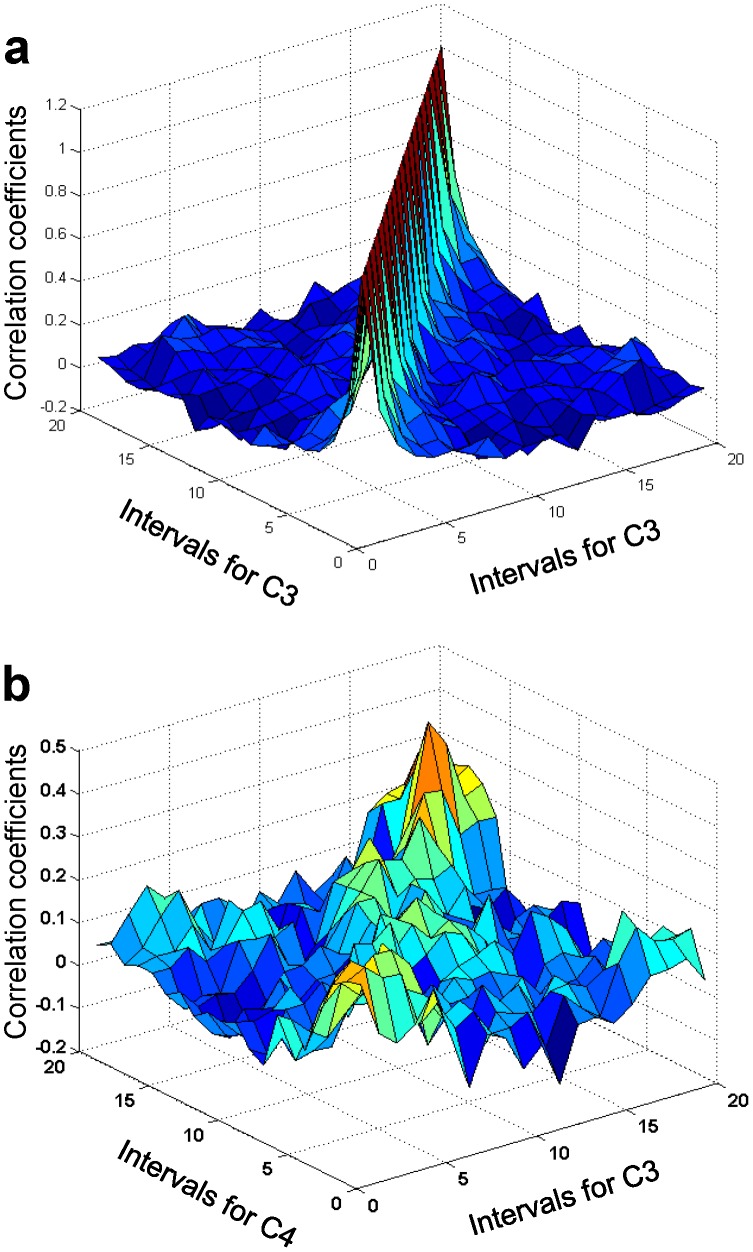
EEG/EEG correlation function. (a) Auto-correlation function for channel C3. (b) Cross-correlation function for channels C3 and C4.

Finally, we studied the correlation function 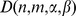 dependent on two times, or two sets of time intervals (**Figure 16**). In sharp contrast to the case of muscle activity (**Figure 7** and **Figure 8**), we did not find any long-time correlations between EEG signals recorded over the motor cortex. The characteristic correlation time for a single channel was of the order of 100 ms or less, as seen from **Figure 16a**, where the correlation function 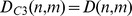 for channel C3 is plotted.

Moreover, we did not find long-time correlations between channels lying within the left, right, or central motor cortex sites. The characteristic correlation time in this case, which corresponded to the characteristic time of cross-talk between channels, was also on the order of 100 ms or less. More accurate computations of the characteristic correlation times would require a more precise trial alignment, which in our experiments was within several tens of milliseconds.

Finally, we found that correlations between channels that overlie different parts of motor cortex, e.g., channel C3 (the left part) and channel C4 (the right part) (**Figure 16b**), were strongest at coincident time intervals and quickly became insignificant as the time interval between recorded events increased. Thus, there were no significant long-time correlations between channels among different parts of the motor cortex.

### 5. Correlation functions of EMG and EEG signals

**Figure 17:**
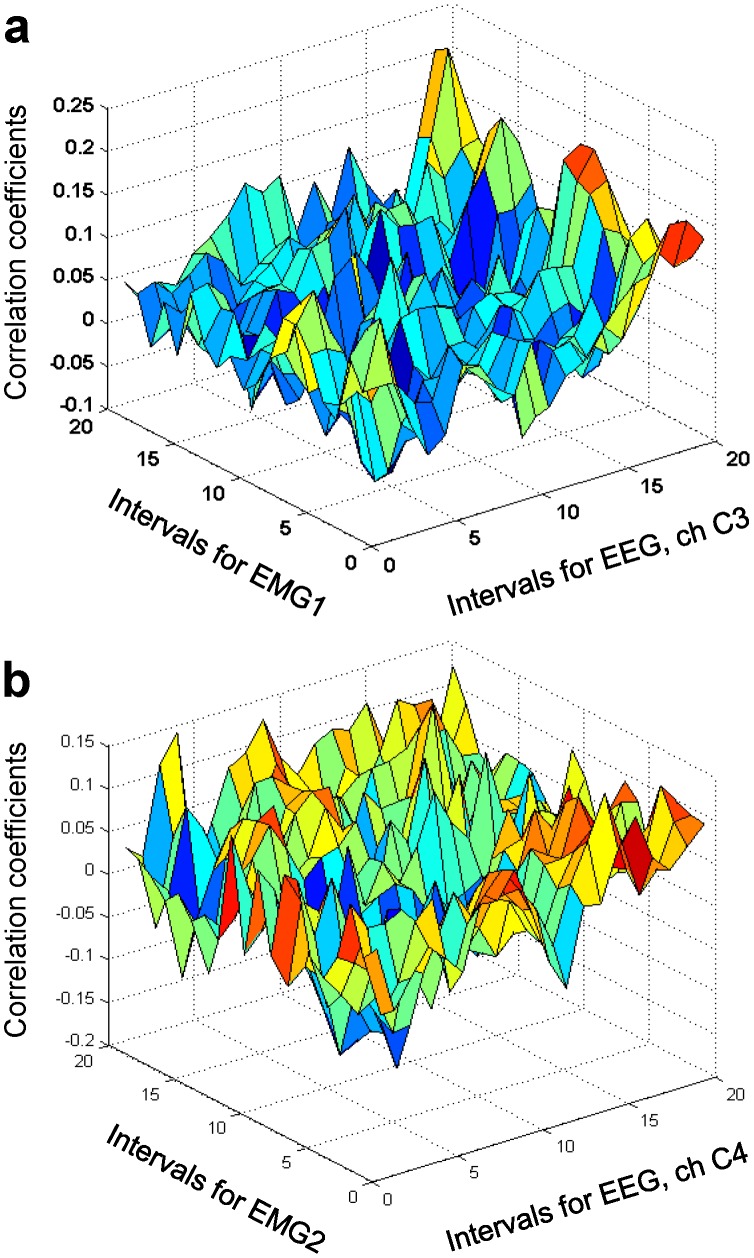
EEG/EMG correlation functions. (a) Correlation function for EMG channel 1 and EEG channel C3. (b) Correlation function for EMG channel 2 and EEG channel C4.

Since the logarithms of both EMG energy and EEG energy in motor cortex channels were normally distributed over trials, we computed the correlation functions between EMG and EEG signals as
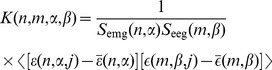
(18)which detects simultaneous trial-to-trial variations of EEG activity and EMG activity (**Figure 17**). As in the case of EEG/EEG correlation functions, we restricted our computations to the 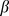 spectral range of EEG signals.

The energy of EMG signals grew in the time period shortly before and during the pen-on-paper period (**Figure 3a and 3b**), while the energy of EEG signals in the motor cortex channels decreased during the same time period (**Figure 9b**). Therefore, we expected to find an *anticorrelation* between EMG and EEG signals, i.e. the correlation function 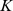 would be negative in the time intervals shortly before and during the pen-on-paper period.

**Figure 18:**
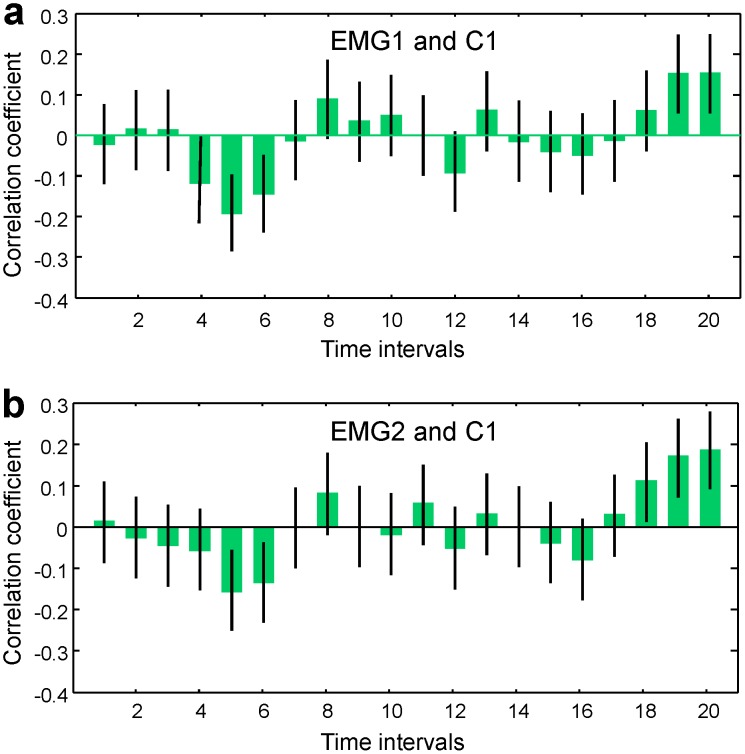
EEG/EMG correlation functions at coincident time intervals. The correlation functions for EEG channel C1 and EMG channels 1 (a) and 2 (b). Error bars indicate 95% confidence bounds. The values of the correlation coefficient are statistically significant if the upper and lower confidence limits have the same sign.

Although correlation coefficients at coincident time intervals, shown in **Figure 18**, were statistically significant at some time intervals, they were smaller than EEG/EEG and EMG/EMG correlation coefficients. When statistically significant EMG/EEG correlations occurred, they were inconsistent among subjects.

The absence of strong correlations between EMG and EEG signals may indicate that strong trial-to-trial variability may be in part introduced by spinal motoneuron activity, which results in wide dispersion of EMG signals with respect to EEG signals. Additionally, EEG components unrelated to motor output may decrease correlations between EEG and EMG signals.

## Discussion

To derive a dynamical picture of neural activity and of functional relationships between different neural regions, we examined time-dependent statistical and correlation properties of EMG and EEG signals recorded simultaneously during handwriting of digit “3” by 7 subjects. We recorded signals in approximately 400 2000-ms trials during which each subject performed an identical handwriting task. The trials started 1000 ms before the moment of time when the pen touched paper the first time and finished 1000 ms after this moment of time. To study the time dependence of neural signals, the trials were divided into 20 100-ms time intervals.

We studied trial-to-trial variability of EMG signals and EEG signals derived from the motor cortex during the 2000-ms time interval during which subjects performed the same handwriting task. We found that the trial-to-trial distribution of the neural signal energy was described well by a log-normal distribution and not by a normal distribution. Whereas the distribution parameters - the mean value and the dispersion - depended on intra-trial time, the log-normal distribution was found for all of 20 time intervals.

We computed the Pearson auto- and cross-correlation functions for EMG signals and EEG signals recorded from the motor cortex. We observed very strong correlations at coincident time intervals between EMG signals recorded from different muscle groups. Moreover, these correlations were long-time and remained quite strong during almost the entire 2000-ms time period.

We also found strong correlations between EEG signals in 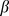 spectral range, provided that channel pairs were located in the motor cortex of the same hemisphere. The correlations between signals recorded from different sides of the skull were much weaker. Moreover, in contrast to the EMG/EMG correlations, correlations between EEG signals existed only over short durations that did not exceed 100 ms.

Finally, we found cross-correlation functions between EEG and EMG signals. We suggest that low correlation coefficients between EEG and EMG activities may be explained by an abundance of EEG signals unrelated to low-level motor parameters and by non-cortical sources of a trial-to-trail variability of spinal motoneuron activity even though the subjects performed a stereotypical handwriting task.
